# Exploring stability-based voxel selection methods in MVPA using cognitive neuroimaging data: a comprehensive study

**DOI:** 10.1007/s40708-016-0048-0

**Published:** 2016-04-06

**Authors:** Miaolin Fan, Chun-An Chou

**Affiliations:** Binghamton University, the State University of New York, 4400 Vestal Pkwy E, Binghamton, NY 13902 USA

**Keywords:** Feature selection, Stability, Functional MRI, Multi-voxel pattern analysis

## Abstract

Feature selection plays a key role in multi-voxel pattern analysis because functional magnetic resonance imaging data are typically noisy, sparse, and high-dimensional. Although the conventional evaluation criterion is the classification accuracy, selecting a stable feature set that is not sensitive to the variance in dataset may provide more scientific insights. In this study, we aim to investigate the stability of feature selection methods and test the stability-based feature selection scheme on two benchmark datasets. Top-k feature selection with a ranking score of mutual information and correlation, recursive feature elimination integrated with support vector machine, and L1 and L2-norm regularizations were adapted to a bootstrapped stability selection framework, and the selected algorithms were compared based on both accuracy and stability scores. The results indicate that regularization-based methods are generally more stable in StarPlus dataset, but in Haxby dataset they failed to perform as well as others.

## Introduction

**Fig. 1:**
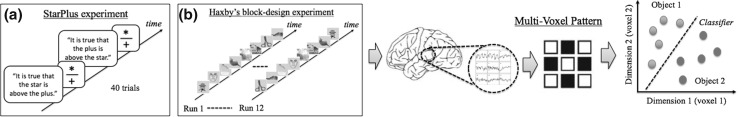
A demonstration of MVPA of fMRI data in cognitive experiments. Visual stimuli are presented to subjects during experiment tests and fMRI data are collected over time. Informative voxels are identified as a pattern used for classification among visual stimuli

Exploring the mysteries of brain function is one of the most challenging and fascinating tasks in the domain of science. In recent years, with the advent of machine learning techniques, the interdisciplinary field of machine learning and neuroscience has drawn growing attention to both communities. With the aid of modern neuroimaging techniques, the capability of machine learning algorithms to identify distributed patterns of voxels in response to stimuli allows for decoding brain activities using data-driven models. A comprehensive review of previous studies has been provided in [1–3]. In this study, we would like to focus on multi-voxel pattern analysis (MVPA) [4], which is a commonly used methodological framework for analyzing functional magnetic resonance imaging (fMRI) data with machine learning algorithms (see Fig. 1). fMRI is a popular, non-invasive neuroimaging technique to measure brain activity via blood-oxygen-level dependent (BOLD) signals, recorded as time series in a three-dimensional (3D) brain space. The precise spatial localization of brain activation, therefore, is an essential advantage of fMRI compared to other non-invasive neuroimaging techniques. Unlike conventional univariate approaches, MVPA constructs a pattern classification problem to decode neural information processing by characterizing multivariate brain activity patterns [5].

However, fMRI-based data analysis using machine learning approaches has a challenging small-*n* large-*p* problem, i.e., there are many thousands of voxels in the brain, but the sample size is relatively small because of the expensive cost of fMRI data collection. Moreover, only a portion of the brain will be activated with respect to specific stimulus or mental states. Hence, selecting the active voxels associated with particular stimuli or states is an important and challenging task before training classifiers in MVPA, which is called *feature selection* or *feature reduction*. In current studies, a common criterion of evaluating the subset selection is classification accuracy. This evaluation criterion may suffer from the variance in training data with a limited sample size and result in unstable generalization error when the trained model is applied to an unknown dataset. Selecting stable features across various datasets, on the other hand, has not been completely investigated. Therefore, the objective of our study is to explore for an integrated stability-based feature selection approach.

The remainder of this paper is organized as follows: Section 2 provides a brief review of existing studies, including stability selection algorithms and their applications to neuroimaging data. Section 3 illustrates the methodology, including experimental settings, data description, feature extraction and selection methods, classification algorithms, and methodological framework. Results are reported and discussed in Sect. 4, followed by the conclusion and possible directions for future work in Sect. 5.

## Literature review

A major challenge in MVPA, as stated previously, comes from the high dimensionality and sparsity in fMRI data. Hence, the regularized logistic regression (LR) such as least absolute shrinkage and selection operator (LASSO) and elastic net (or ENet for short) are found to be particularly useful in addressing sparsity. Another general objective of feature selection is to build interpretable models which are able to support or reject hypothesis with domain knowledge. To this end, selecting a stable subset that is robust to the variance in samples is of great importance. Numerous studies have discussed the stability issue using various types of feature selection methods from statistician’s perspective [6–9]. Numerous metrics to quantify the stability in feature selection were proposed, but no standard guideline for comparing various feature selection methods has been acknowledged up to date [6, 7, 10, 11]. In this section, a brief review of existing studies of stability selection is provided in terms of methodology and applications to neuroimaging data.

Before Meinshausen and Buhlmann [8] proposed their methodological framework of stability selection, some early studies have implied the usefulness of re-sampling strategy such as bootstrap of improving the stability of feature selection [7, 12]. In Meinshausen and Buhlmann’s work, the subset selection is performed via repeatedly running LASSO on re-sampled subsets, while each subset is half the size of original samples. A feature is able to enter the model only if the frequency of being selected is greater than a user-defined threshold (denoted as $\varTheta$ below). This method was later improved in [9] by changing the re-sampling mechanism such that if one half of the dataset was sampled, the other complimentary half should also be used. This Complimentary Pairs Stability Selection (CPSS) method has been mathematically proved to provide an improved bound for the estimation error control. An interesting aspect of stability selection is that although original stability selection approach was claimed not to be sensitive to the selection of $\varTheta$ in a range of [0.6, 0.9], it was reported in the CPSS article [9] that the choice of $\varTheta$ may have an impact. In general, stability selection is a topic that has not been fully discovered.

Stability-based data analysis approaches based on neuroimaging data have drawn growing interest from neuroscientists in recent years, and have been widely adopted as a methodological framework in existing studies. The great potential of stability selection lies in its adaptability, which allows users to develop their own approaches with various focuses as well as domain knowledge in order to construct more powerful knowledge discovery systems. The existing applications are limited in quantity, but rich in diversity from the following aspects. First of all, in current studies, stability selection has been used to satisfy a variety of research purposes such as exploring the brain functionality in visual pathways [11], functional networks [13, 14], resting-state networks [15], or the localization (or identification) of significant biomarkers relevant to specific mental states [16] or diagnose brain-related disorders [17]. Second, in terms of methodology, numerous variations were made utilizing the concept of stability selection. For example, the possible features for stability selection can be extracted from the functional network; in addition to voxels (or nodes in network science), selecting discriminative connectivity (edges) is also helpful to understand the mechanism underlying functional networks [13, 14, 18, 19]. Moreover, some studies integrated other machine learning algorithms such as clustering [15, 20, 21], graphical lasso [18], and support vector machine (SVM) [14]. A novel variation of LASSO was proposed to search for similar but not identical voxels in feature selection across multiple human subjects [22]. Finally, although the original stability selection was proposed as a data-driven model, some novel methods also utilized anatomical information, topological structure, or other structural information underlying features to enhance its stability and predictive power [16, 23].

## Methodology

### Data description

Two benchmark datasets in cognitive science were used in our study: (1) StarPlus dataset [24] and (2) Haxby dataset [4].

#### StarPlus dataset

This dataset is named StarPlus because of the visual stimuli presented to subjects during the experiments. Subjects were instructed to focus on the visual stimulus on the screen when fMRI data was recorded. In one half of all experiment trials, a sentence (semantic stimulus) was presented first for 4 s (e.g., “It is true that the star is above the plus.”), followed by an image (symbolic stimulus) showing similar information for another 4 s (see Fig. 1a). The subjects need to press a button to indicate whether if the sentence and image matches each other. In remaining trials, the sequence of presenting sentences and images switches. 40 trials were conducted during this experiment, each of which contains 2 samples labeled by the type of stimulus (semantic = ‘0,’ symbolic = ‘1’).

The fMRI data was collected at 500 ms sampling rate in a 3D space of $64 \times 64 \times 8$ voxels, and the pre-processed data of 6 subjects is available to public. The scanned area contains 25–30 anatomical regions of interest (ROIs), which have approximately 4000 voxels. Particularly, 7 ROIs are highlighted by the proposer as they are most relevant to this task. Thus, the number of voxels to be analyzed in our study is reduced to around 2000, varying from subject to subject.

#### Haxby dataset

Haxby dataset contains the fMRI scans of 6 subjects. The experiment has 12 trials, each of which lasts for about 24 s, separated by rest periods (see Fig. 1b). In each trial, 8 images presenting 8 types of objects including houses, human faces, cats, and so on. Images were shown on the screen for 500 ms of each; the inter-stimulus interval is 1500 ms. The entire experiment was then partitioned into $12\times 8 = 96$ samples from each individual with only one trial removed from subject 5 who was corrupted during this trial. The fMRI scans were collected in a space of $40 \times 64 \times 64$ voxels, corresponding to a voxel size of $3.5 \times 3.75 \times 3.75$ mm$^{3}$, and a volume repetition time of 2.5 s [4]. Similarly, instead of examining the whole brain, our study is focused on the visual cortex area which consists of up to 675 voxels based on the anatomical information of our subjects.

### Feature extraction

**Fig. 2:**
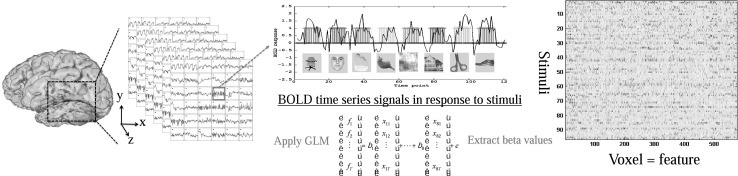
The feature (beta values) matrix is extracted by applying a general linear model to fMRI BOLD signals. Subject 1 in Haxby dataset is used as an illustrative example

General linear model (GLM) approach as introduced in [25] was applied to the time series data for feature extraction. The basic concept is to characterize BOLD signals by fitting GLM to a haemodynamic response function (HRF) that describes blood-oxygen-level responses to the given stimulus as a function of time. The estimates of the coefficients $\hat{\beta } = \lbrace \beta _1, ... \beta _m \rbrace ^T$ in GLM model: $Y = X\beta$ represent the time-related response of each individual voxel to the stimulus of interest. Using $\hat{\beta }$ as features results in an m-dimensional feature space, where each voxel is represented by its beta value $\hat{\beta }_j, j \in \lbrace 1, ..., m \rbrace$. In our study, pre-processing and feature extraction were implemented in Matlab 8.3 [26] using a toolbox [27]. Figure 2 illustrates extracting beta values as features for subject 1 in the Haxby dataset, where the samples (stimuli) are ordered in the same sequence as presented in the experiment.

### Feature selection

Current feature selection methods are categorized into three classes based on how the subset-search algorithm is combined with the classification procedure: filter, wrapper, and embedded [28, 29]. In this subsection, the selected feature selection methods are reviewed under this framework.

#### Filter approach

Filter methods are relatively independent of classification comparing to other types of methods, and the interactions among features are completely ignored. With a pre-defined metric of relevance between individual features and class labels, all features are ranked and the top-n features comprise the subset selection. In this study, Pearson correlation (referred to as Corr) and mutual information (MI) were employed as they are commonly used metrics. Moreover, the size of subset to be selected is not arbitrarily determined, but optimized using a cross-validation scheme. Since the classifier used in combination with these filter methods is SVM, these approaches will be referred to as SVM-MI, SVM-Corr in the following sections.

#### Wrapper approach

Instead of evaluating the similarity between individual features and class labels, wrapper methods seek for a best subset of features by evaluating the subset as a whole based on classification performance. The recursive feature elimination (RFE) integrated with SVM, referred to as SVM-RFE, was chosen as an example of wrapper approach in our study. It is a backward feature selection approach which starts with the entire feature set and iteratively removes a proportion of features after evaluation using SVM, which was implemented using a toolbox in Matlab 8.3 [30]. However, a significant disadvantage of wrapper methods is the computational cost: the classification algorithm need to be performed repeatedly for every subset in the candidate pool, which will largely increase the computational time especially with high-dimensional data. In order to be consistent with filter methods, the size of subset in feature selection was also optimized using a cross-validation scheme.

#### Embedded approach

The embedded methods utilize regression models with regularization. In such models, the feature selection is embedded in the training process of classification algorithm by optimizing a penalty parameter $\lambda$. With an appropriate $\lambda$ selected using a cross-validation scheme, all redundant features are removed from the model by forcing their coefficients to be zero. In this study, we employ both LASSO and ENet as embedded approaches. More details about these algorithms are to be discussed later in Sect. 3.4.

### Classification algorithms

Consider that in a binary classification problem, the input data are a set of data points $X = \lbrace x_1,...,x_n \rbrace$ in an *m*-dimensional feature space, i.e., $x_i \in \, R^m \, \forall \,i\,\in \lbrace 1,...,n \rbrace$, where *n* is the number of data points and *m* is the number of features. The corresponding target values $T = \lbrace t_1,...,t_n \rbrace$ are the class labels. The predictions of class labels are denoted by $Y = \lbrace y_1,...,y_n \rbrace$. The objective of classification algorithms is to estimate the optimal parameters *w* and *b*, such that the mapping $f: X \rightarrow Y$ best captures the relationship between inputs and targets.

#### Support vector machine

SVM is a classifier that optimizes the decision boundary with a maximum geometrical margin, i.e., the distance between decision boundary and the closest data points in each class. The soft-margin SVM with a linear kernel is formulated as follows:

1 $$\arg \, \min _{w,b} \,\frac{1}{2}\Vert w\Vert ^2 + C \sum ^n_{i=1}(\xi _i),$$

2 $$s.t.\quad t_i(w^Tx_i+b) \ge 1 - \xi _i \quad \forall ~ i \in \lbrace 1,..., n \rbrace,$$

3 $$\xi _i \ge 0 \quad \forall \, i \in \lbrace 1,..., n \rbrace ,$$

where slack variables $\xi _i$ are introduced to give tolerance to the misclassified data points lying in between support vectors, parameter *C* controls the tolerance level, and the target values $t_i \in \lbrace {-1,1}\rbrace$. The decision boundary of a linear classifier is a hyperplane described by the function: $f(x) = w^Tx + b$, therefore for any data point *i* if $f(x_i)>0, \,y_i = 1$; otherwise $\,y_i = -1$.

#### Regularized logistic regression

LASSO is a shrinkage method proposed by Tibshirani [31], which is applicable to both linear and logistic regression models; ENet is a widely used variation of LASSO proposed by Zou and Hastie [32]. In linear regression, the objective of LASSO is to find optimal solution for the following problem:

4 $$\arg \, \min _{w,b} \,\sum \vert f(x_i) - t_i \vert ^2,$$

5 $$s.t.\sum |w| \le \lambda,$$

where $\lambda$ is a tuning parameter which controls the shrinkage. This formulation can be generalized to logistic regression models by replacing Eq. (4) with the cost function in LR model. Similarly, the formulation of ENet shares the same objective function as in Eq. (4) but the constraint is as follows:

6 $$s.t.(1 - \alpha ) \sum |w| + \alpha \sum \vert w \vert ^2 \le \lambda,$$

where $\alpha$ controls the trade-off between ridge regression and LASSO. In our study, $\alpha = 0.8$ is used as a common selection.

### Methodological framework

Although the concepts of stability selection were utilized in this study, the setup of experiment differs in two datasets. Table 1 presents the cross-validation settings of both datasets. Let $D^T$, $D{^G},$ and $D^V$ denote training, test and validation set, respectively. The general framework is demonstrated as follows:

- Step 1:    Randomly take a subset $D^S$ out of training set $D^T$;
- Step 2:    Run the feature selection method on set $D^S$ while using $D^V$ to control the tuning parameters in selected algorithm;
- Step 3:    Repeat step 1 and 2 *n* times;
- Step 4:    Use a set of most frequently selected features *S* as the future feature set;
- Step 5:    Train the model with selected features on $D^T$ and $D^V$;
- Step 6:    Evaluate the performance on $D^G$;
- Step 7:    Repeatedly perform Step 1 to 6 according to selected cross-validation scheme.

In Step 2, after specifying the $D^T$, $D{^G},$ and $D^V$, the re-sampling was performed 50 times on $D^T$ for feature selection with simultaneous validation on $D^V$. Provided that stability selection method proposed a re-sampling scheme with embedded feature selection methods [8, 9], our approach was designed to utilize the filter and wrapper methods in the same manner such that the results can be compared apples to apples. Further, ten replications were conducted based on different settings of $D^T$, $D{^G},$ , and $D^V$ for StarPlus dataset, while twelve replications were performed for Haxby dataset such that each trial was used exactly once as test set.

The stability measure in our study is Jaccard Index [33], a measure of similarity between two sets. Suppose there are two subsets $S_a$ and $S_b$, then the Jaccard Index for ($S_a, S_b$) is defined as

7 $$J_C(S_a, S_b) = \frac{\mid S_a \cap S_b \mid }{\mid S_a \cup S_b\mid },$$

where $\mid S \mid$ is the number of elements in set *S*.

When there are *k* subsets, the overall similarity is computed by averaging the pairwise Jaccard Index for all possible pairs. The formulation is given as follows:

8 $$J_{C_k} = \frac{2}{k(k-1)} \sum _a \sum _{b \ne a}J_C(S_a, S_b).$$

**Table 1:** The cross-validation settings of datasets

Dataset	Training	Test	Validation	Replication
StarPlus	60	10	10	10
Haxby	6	5	1	12

Note that StarPlus dataset is measured in samples, while the Haxby dataset is measured in trials

## Results and discussions

In this section, the results are presented and discussed from the following aspects. First, a comparison among selected feature selection methods is provided based on accuracy and stability. Second, the selection of $\varTheta$ is further examined to provide some suggestions for future studies. Finally, the localization of voxels selected by each method is discussed to provide some insights.

### Feature selection methods

As shown in Tables 2–13, the classification performance has a large variance across algorithms and subjects. In this section, some discussions are separately given to two datasets since algorithms performed differently in our experiment.

#### Filter and wrapper methods

In StarPlus dataset, SVM-MI, SVM-RFE, and SVM-Corr performed at a comparable level as embedded algorithms in terms of accuracy, but embedded algorithms yielded a better overall stability. Moreover, it is not desirable that SVM-Corr sometimes selected a large subset although it is always highly stable. It may imply that SVM-Corr approach, according to the current experiment settings, tends to overfit in some cases. In Haxby data, however, SVM-MI, SVM-RFE, and SVM-Corr algorithms are more accurate than embedded algorithms in general. In terms of computational cost, SVM-MI and SVM-Corr are much faster than SVM-RFE. Among these three algorithms, SVM-MI is suggested based on overall accuracy, stability, and computational time, which is interestingly consistent with a previous study using same dataset without utilizing stability selection [34].

#### Embedded methods

In general, ENet has higher stability and standard deviation compared to LASSO; also, it selects a larger and more stable subset. It indicates that throughout all replications the ENet has more stable subsets in *feature selection*, but these subsets yielded an unstable *predictive power* compared to LASSO. Comparison based on the best performing model, ENet yields better accuracy than LASSO in general, which is also supported by previous study using the same dataset [35]. This phenomenon may relate to the balance between *variance* and *bias* of generalization error in statistics. The stability selection scheme provides a control to help avoid the situation of having an unstable feature subset in the model. On the other side, however, by reducing the total number of available samples for training purposes, it seems to scarify accuracy to some extent. This raises questions that, if it is possible to design a systematic approach to achieve or control the balance between stability and accuracy. Depending on the objective of their studies, some researchers may favor an interpretable model to explore or support a hypothesis, while others may prefer a predictive one for practical use.

**Fig. 3:**
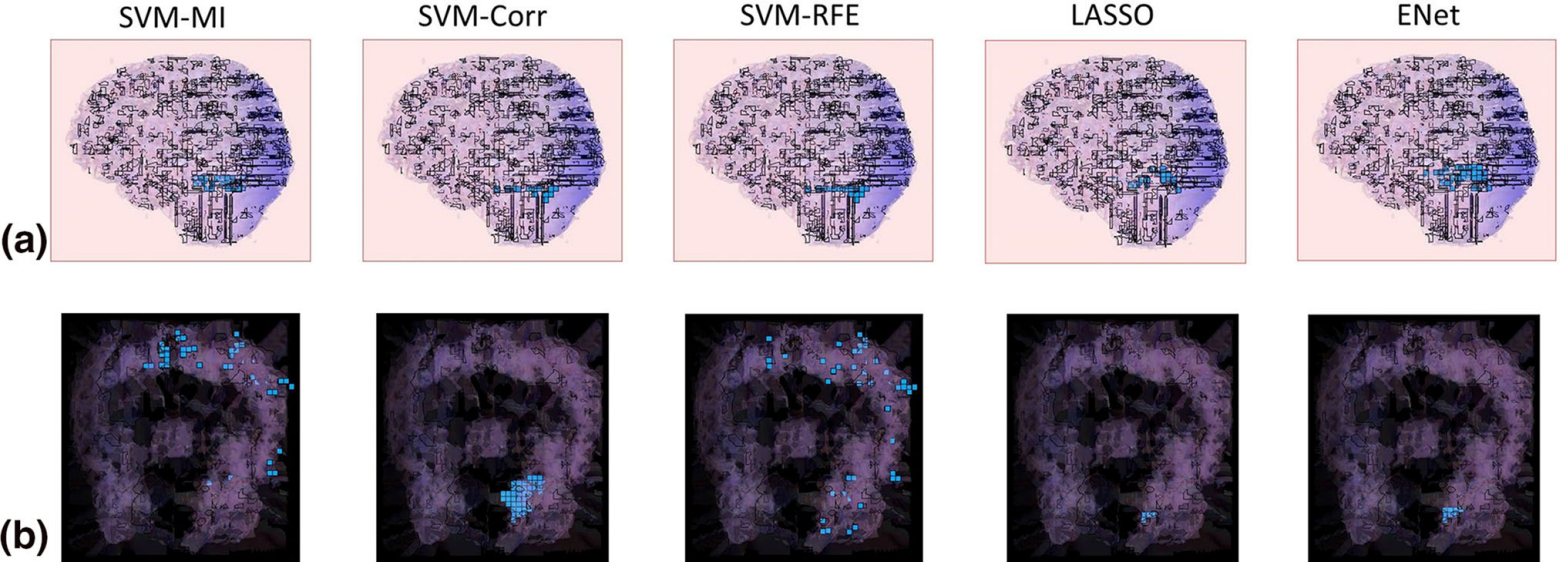
An illustration of the distribution of voxels selected by each method in the visual cortex area for (**a**) subject 1 in Haxby dataset and (**b**) subject 04820 in StarPlus dataset

### Threshold selection

According to the our experimental results, the selection of $\varTheta$ within [0.6, 0.9] has a significant influence on classification accuracy. This finding is consistent with the comments in [9]. More interestingly, a rough trend seems to imply that LASSO favors a smaller $\varTheta$ while ENet prefers a larger one. As no previous studies have reported this behavior in stability selection based on our knowledge, we can only make intuitive inference for the possible reason. Since group effect is encouraged in ENet, it tends to introduce more features into the model than LASSO, and thus a higher $\varTheta$ is preferred to avoid introducing too many redundant features. Another interesting observation is the correlation to stability scores. For most subjects, the stability scores seem to be negatively correlated with $\varTheta$ in LASSO and ENet, which indicates setting up a high threshold may have a negative impact on model stability.

The size of subset to be selected after re-sampling and replications, however, does not show any correlations with $\varTheta$ in stability selection. Moreover, the size of subset remains stable in general for the same subject with a varying $\varTheta$. These findings encourage further exploration for standard guidelines for the selection of $\varTheta$ with empirical or theoretical supports.

### Voxel selection and visualization

Figure 3 presents a visualization of selected voxels for subject 1 in Haxby dataset and subject 04820 in StarPlus dataset. Subset selection is determined by picking up the most stable voxels, namely, the voxels with highest selection frequency throughout all replications. In general, the algorithms with higher stability scores: SVM-Corr, LASSO, and ENet selected a cluster of voxels located in visual cortex area, which is consistent with the domain-specific knowledge, while SVM-MI and SVM-RFE had a sparse voxel distribution. This indicates that stability-based feature selection framework provides a more stable, interpretable subset selection, which is difficult to achieve by evaluating models using accuracy.

**Table 2:** Summary of results—subject 04799 in StarPlus dataset

Method	Mean accuracy (%)	STD (%)	Average number of selected features	Stability
SVM-MI	50.00	17.00	56	0.32
SVM-Corr	46.00	16.47	51	0.32
SVM-RFE	55.00	17.16	70	0.40
LASSO ($\varTheta$ = 0.6)	41.00	12.87	6	0.16
LASSO ($\varTheta$ = 0.7)	48.00	11.35	4	0.23
LASSO ($\varTheta$ = 0.8)	48.00	11.35	7	0.36
LASSO ($\varTheta$ = 0.9)	49.00	3.16	6	0.06
ENet ($\varTheta$ = 0.6)	43.00	13.37	10	0.20
ENet ($\varTheta$ = 0.7)	45.00	15.81	6	0.21
ENet ($\varTheta$ = 0.8)	48.00	13.17	8	0.41
ENet ($\varTheta$ = 0.9)	47.00	10.59	5	0.10

**Table 3:** Summary of results—subject 04820 in StarPlus dataset

Method	Mean accuracy (%)	STD (%)	Average number of selected features	Stability
SVM-MI	90.00	10.54	164	0.40
SVM-Corr	83.00	15.67	1845	0.98
SVM-RFE	91.00	11.01	127	0.34
LASSO ($\varTheta$ = 0.6)	85.00	8.50	8	0.78
LASSO ($\varTheta$ = 0.7)	85.00	8.50	8	0.71
LASSO ($\varTheta$ = 0.8)	84.00	8.43	10	0.49
LASSO ($\varTheta$ = 0.9)	73.00	14.94	6	0.24
ENet ($\varTheta$ = 0.6)	85.00	10.80	14	0.64
ENet ($\varTheta$ = 0.7)	85.00	10.80	12	0.71
ENet ($\varTheta$ = 0.8)	85.00	10.80	15	0.92
ENet ($\varTheta$ = 0.9)	86.00	8.43	14	0.89

**Table 4:** Summary of results—subject 04847 in StarPlus dataset

Method	Mean accuracy (%)	STD (%)	Average number of selected features	Stability
SVM-MI	80.00	4.71	64	0.59
SVM-Corr	82.00	10.33	1660	0.97
SVM-RFE	83.00	9.49	50	0.39
LASSO ($\varTheta$ = 0.6)	77.00	8.23	4	0.60
LASSO ($\varTheta$ = 0.7)	76.00	8.43	7	0.69
LASSO ($\varTheta$ = 0.8)	79.00	9.94	5	0.82
LASSO ($\varTheta$ = 0.9)	79.00	9.94	5	0.90
ENet ($\varTheta$ = 0.6)	77.00	11.60	7	0.46
ENet ($\varTheta$ = 0.7)	78.00	10.33	13	0.53
ENet ($\varTheta$ = 0.8)	77.00	8.23	9	0.56
ENet ($\varTheta$ = 0.9)	80.00	9.43	13	0.69

**Table 5:** Summary of results—subject 05675 in StarPlus dataset

Method	Mean accuracy (%)	STD (%)	Average number of selected features	Stability
SVM-MI	87.00	6.75	70	0.47
SVM-Corr	78.00	11.35	2059	0.88
SVM-RFE	90.00	8.16	50	0.39
LASSO ($\varTheta$ = 0.6)	89.00	7.38	11	0.60
LASSO ($\varTheta$ = 0.7)	87.00	10.59	11	0.54
LASSO ($\varTheta$ = 0.8)	86.00	9.66	11	0.54
LASSO ($\varTheta$ = 0.9)	82.00	9.19	18	0.61
ENet ($\varTheta$ = 0.6)	90.00	6.67	25	0.75
ENet ($\varTheta$ = 0.7)	88.00	10.33	19	0.73
ENet ($\varTheta$ = 0.8)	85.00	8.50	25	0.61
ENet ($\varTheta$ = 0.9)	82.00	10.33	23	0.60

**Table 6:** Summary of results—subject 05680 in StarPlus dataset

Method	Mean accuracy (%)	STD (%)	Average number of selected features	Stability
SVM-MI	74.00	8.43	85	0.52
SVM-Corr	73.00	14.18	2211	0.99
SVM-RFE	75.00	15.81	298	0.19
LASSO ($\varTheta$ = 0.6)	80.00	8.16	4	1.00
LASSO ($\varTheta$ = 0.7)	80.00	8.16	4	1.00
LASSO ($\varTheta$ = 0.8)	80.00	8.16	4	1.00
LASSO ($\varTheta$ = 0.9)	80.00	8.16	4	1.00
ENet ($\varTheta$ = 0.6)	79.00	7.38	6	0.82
ENet ($\varTheta$ = 0.7)	78.00	7.89	9	0.84
ENet ($\varTheta$ = 0.8)	80.00	8.16	8	0.76
ENet ($\varTheta$ = 0.9)	80.00	8.16	8	0.72

**Table 7:** Summary of results—subject 05710 in StarPlus dataset

Method	Mean accuracy (%)	STD (%)	Average number of selected features	Stability
SVM-MI	83.00	9.49	52	0.54
SVM-Corr	70.00	6.67	1861	0.99
SVM-RFE	76.00	10.75	93	0.27
LASSO ($\varTheta$ = 0.6)	88.00	13.17	10	0.76
LASSO ($\varTheta$ = 0.7)	86.00	12.65	8	0.64
LASSO ($\varTheta$ = 0.8)	84.00	12.65	9	0.68
LASSO ($\varTheta$ = 0.9)	79.00	11.01	8	0.71
ENet ($\varTheta$ = 0.6)	91.00	8.76	12	0.78
ENet ($\varTheta$ = 0.7)	90.00	9.43	13	0.87
ENet ($\varTheta$ = 0.8)	86.00	12.65	11	0.78
ENet ($\varTheta$ = 0.9)	86.00	12.65	12	0.66

**Table 8:** Summary of results—subject 1 in Haxby dataset

Method	Mean accuracy (%)	STD (%)	Average number of selected features	Stability
SVM-MI	90.63	12.07	122	0.70
SVM-Corr	84.38	16.96	338	0.58
SVM-RFE	84.38	16.96	219	0.49
LASSO ($\varTheta$ = 0.6)	79.17	14.43	88	0.68
LASSO ($\varTheta$ = 0.7)	77.08	13.93	75	0.67
LASSO ($\varTheta$ = 0.8)	76.04	11.25	87	0.71
LASSO ($\varTheta$ = 0.9)	71.88	16.10	95	0.61
ENet ($\varTheta$ = 0.6)	38.54	26.36	255	0.71
ENet ($\varTheta$ = 0.7)	59.38	20.03	235	0.70
ENet ($\varTheta$ = 0.8)	62.50	21.98	255	0.67
ENet ($\varTheta$ = 0.9)	80.21	11.25	232	0.67

**Table 9:** Summary of results—subject 2 in Haxby dataset

Method	Mean accuracy (%)	STD (%)	Average number of selected features	Stability
SVM-MI	70.83	13.41	123	0.57
SVM-Corr	71.88	12.07	357	0.86
SVM-RFE	78.13	14.23	195	0.67
LASSO ($\varTheta$ = 0.6)	55.21	6.44	94	0.57
LASSO ($\varTheta$ = 0.7)	48.96	17.24	90	0.52
LASSO ($\varTheta$ = 0.8)	48.96	17.24	97	0.46
LASSO ($\varTheta$ = 0.9)	43.75	12.50	104	0.46
ENet ($\varTheta$ = 0.6)	32.29	16.39	269	0.64
ENet ($\varTheta$ = 0.7)	50.00	18.46	264	0.62
ENet ($\varTheta$ = 0.8)	53.13	22.06	214	0.58
ENet ($\varTheta$ = 0.9)	47.92	12.87	252	0.55

**Table 10:** Summary of results—subject 3 in Haxby dataset

Method	Mean accuracy (%)	STD (%)	Average number of selected features	Stability
SVM-MI	82.29	18.04	195	0.78
SVM-Corr	80.21	22.27	260	0.87
SVM-RFE	85.42	13.93	157	0.66
LASSO ($\varTheta$ = 0.6)	68.75	14.60	75	0.60
LASSO ($\varTheta$ = 0.7)	71.88	19.31	80	0.57
LASSO ($\varTheta$ = 0.8)	64.58	18.34	79	0.58
LASSO ($\varTheta$ = 0.9)	60.42	19.09	70	0.57
ENet ($\varTheta$ = 0.6)	40.63	17.78	242	0.71
ENet ($\varTheta$ = 0.7)	61.46	18.04	276	0.67
ENet ($\varTheta$ = 0.8)	62.50	15.08	263	0.64
ENet ($\varTheta$ = 0.9)	62.50	10.66	231	0.62

**Table 11:** Summary of results—subject 4 in Haxby dataset

Method	Mean accuracy (%)	STD (%)	Average number of selected features	Stability
SVM-MI	68.75	12.50	58	0.58
SVM-Corr	71.88	17.78	141	0.77
SVM-RFE	71.88	14.23	188	0.56
LASSO ($\varTheta$ = 0.6)	56.25	22.30	30	0.52
LASSO ($\varTheta$ = 0.7)	45.83	21.54	31	0.51
LASSO ($\varTheta$ = 0.8)	42.71	22.90	28	0.36
LASSO ($\varTheta$ = 0.9)	27.08	12.87	34	0.29
ENet ($\varTheta$ = 0.6)	51.04	17.24	136	0.56
ENet ($\varTheta$ = 0.7)	60.42	17.54	132	0.55
ENet ($\varTheta$ = 0.8)	62.50	19.94	149	0.54
ENet ($\varTheta$ = 0.9)	55.21	17.24	124	0.50

**Table 12:** Summary of results—subject 5 in Haxby dataset

Method	Mean accuracy (%)	STD (%)	Average number of selected features	Stability
SVM-MI	64.77	30.53	142	0.62
SVM-Corr	68.18	29.24	255	0.77
SVM-RFE	65.91	29.63	237	0.72
LASSO ($\varTheta$ = 0.6)	51.14	24.01	24	0.58
LASSO ($\varTheta$ = 0.7)	46.59	21.72	23	0.57
LASSO ($\varTheta$ = 0.8)	39.77	22.23	21	0.43
LASSO ($\varTheta$ = 0.9)	15.91	9.83	22	0.12
ENet ($\varTheta$ = 0.6)	45.45	21.12	66	0.54
ENet ($\varTheta$ = 0.7)	39.77	27.28	67	0.52
ENet ($\varTheta$ = 0.8)	46.59	23.78	65	0.53
ENet ($\varTheta$ = 0.9)	48.86	27.07	76	0.59

**Table 13:** Summary of results—subject 6 in Haxby dataset

Method	Mean accuracy (%)	STD (%)	Average number of selected features	Stability
SVM-MI	87.50	10.66	176	0.73
SVM-Corr	79.17	17.94	179	0.73
SVM-RFE	86.46	12.45	279	0.89
LASSO ($\varTheta$ = 0.6)	69.79	17.24	42	0.60
LASSO ($\varTheta$ = 0.7)	62.50	17.68	43	0.59
LASSO ($\varTheta$ = 0.8)	63.54	15.50	39	0.52
LASSO ($\varTheta$ = 0.9)	52.08	14.92	47	0.47
ENet ($\varTheta$ = 0.6)	57.29	18.04	160	0.71
ENet ($\varTheta$ = 0.7)	65.63	17.78	151	0.70
ENet ($\varTheta$ = 0.8)	67.71	15.50	161	0.67
ENet ($\varTheta$ = 0.9)	72.92	12.87	152	0.61

## Conclusion

In this study, we conducted a comprehensive analysis for a selection of filter, wrapper, and embedded feature selection approaches on the two benchmark fMRI datasets, adopting a stability-based methodological framework. It is found that the stability of feature selection is a potential alternative criterion for model selection in addition to classification accuracy, especially for those studies whose objective is to find a model with good interpretation rather than excellent predictive power. Having noticed that it is the case for the majority of neuroimaging data-based studies, developing stability-based feature selection may be helpful for identifying important voxels to decode mental states.

The future studies may explore a reliable metric to quantify the stability of feature selection methods because it has not been clearly defined. A standard guideline for selecting a suitable feature selection approach to achieve higher stability can be developed on the basis of a reliable metric. Also, a methodological framework which enables control of the balance between accuracy and stability is another issue to be further explored. Furthermore, it would be an interesting topic to examine the stability in voxel selection across different subjects, which will also be a challenging task because the activity patterns in brain are known to have large individual variations even in the same cognitive tasks.
